# Exploring Communication Barriers and Facilitators in School Vaccination: A Case Study in South Eastern Sydney, Australia

**DOI:** 10.3390/vaccines12111243

**Published:** 2024-10-31

**Authors:** Leigh McIndoe, Alexandra Young, Cassandra Vujovich-Dunn, Vicky Sheppeard, Stephanie Kean, Michelle Dives, Cristyn Davies

**Affiliations:** 1 South Eastern Sydney Public Health Unit, Sydney, NSW 2031, Australia; leigh.mcindoe@health.nsw.gov.au (L.M.); vicky.sheppeard@health.nsw.gov.au (V.S.); stephanie.kean@health.nsw.gov.au (S.K.); michelle.dives@health.nsw.gov.au (M.D.); 2 Kirby Institute, UNSW Sydney, Sydney, NSW 2052, Australia; 3 School of Public Health, University of Queensland, Brisbane, QLD 4006, Australia; c.vujovichdunn@uq.edu.au; 4 School of Public Health, University of Sydney, Sydney, NSW 2050, Australia; 5 Specialty of Child and Adolescent Health, Faculty of Medicine and Health, University of Sydney, Sydney, NSW 2050, Australia; cristyn.davies@sydney.edu.au; 6 Sydney Infectious Diseases Institute, University of Sydney, Sydney, NSW 2050, Australia

**Keywords:** adolescent vaccination, school-based immunisation, health communication, barriers, facilitators, stakeholders, qualitative methods

## Abstract

**Background/Objectives**: Given the discrepancies in immunisation coverage, the goal of this study was to explore the barriers and facilitators to effective communication across the school-based vaccination program in South Eastern Sydney schools. **Methods**: A qualitative study was undertaken with purposively selected immunisation staff, school coordinators, and parents of Year 7 students who had not received two vaccinations (dTpa and HPV) at school. A focus group with immunisation staff and interviews with school coordinators explored the barriers and facilitators to vaccination uptake, including communication across stakeholders. The parent interviews explored attitudes to vaccination and the school program and investigated the program communication methods. **Results**: Five immunisation staff, eleven school coordinators, and eleven parents participated in the study. The barriers to participation in the school vaccination program included low parent recall of vaccination information, challenges encountered by school staff in consent tracking, no communication channel between health staff and parents, a greater school focus on vaccination facilitation than student education, and limited communication between stakeholders about catch-up vaccinations. The facilitators included established school/parent relationships for vaccine communication, effective communication between health and school staff, and using multiple methods to promote clinic and consent requirements. **Conclusions**: Opportunities exist to increase program participation by enhancing vaccination information and education for students and parents, with better communication about vaccination catch-ups and consent.

## 1. Introduction

School-based immunisation programs are an effective and efficient way to deliver vaccinations to adolescents worldwide [1]. Barriers and facilitators to the school vaccination program are well documented globally [2,3] and include organisational models for vaccine delivery, program leadership, communication and information sharing between key stakeholders, vaccination consent processes, and the catch-up of missed vaccine doses [4]. More recently, evidence suggests that the COVID-19 pandemic has negatively impacted how routine vaccinations are perceived and understood by the community [5,6,7,8].

Communication is an integral part of a successful public health initiative. In terms of vaccination, communication can include written information, educational videos, or vaccine-promoting social media strategies. Parental communication is particularly important for vaccination because parental/guardian (hereafter ‘parental’) consent is required to undertake adolescent vaccination in Australian schools. Improved parent communication strategies about HPV vaccination, for example, can increase HPV knowledge and parental consent numbers [9]. Additionally, targeting young people with high-quality vaccine information and education has also been recommended to increase vaccination knowledge and completion rates [10,11,12,13]. Furthermore, adopting a broader approach that considers not only communication for parents and students but also community values and the organisational and policy environment in which vaccination occurs can impact vaccine acceptance [4,11,14].

Australian states and territories are responsible for the operation of the school-based vaccination program (SBIP) and provide student vaccinations in accordance with the National Immunisation Program (NIP). In the state of New South Wales, local health authorities deliver the school-based program, offering adolescents in Year 7 (12–13 years) the diphtheria, tetanus, and pertussis (dTpa) and human papillomavirus (HPV) vaccinations and students in Year 10 (15–16 years) the meningococcal ACWY vaccine. The Commonwealth government funds these vaccines offered through the SBIP, which are free to eligible adolescents [15].

The South Eastern Sydney Local Health District (SESLHD) covers a region in the southeastern portion of Sydney, Australia, with a population of just under one million people. This region stretches from the central business district of Sydney across seven local government areas. The SESLHD has a diverse population, with 53% of residents born overseas, 38% of households speaking a non-English language, 1% of residents identifying as Aboriginal and/or Torres Strait Islander, and residents from across the full socioeconomic spectrum [16]. The Public Health Unit of the SESLHD manages and conducts the school vaccination program across 96 high schools. An ecological study, with schools as the primary unit of analysis, was conducted in the SESLHD and found discrepancies in the immunisation coverage among schools within the district [17]. This finding is consistent with previous results in NSW and other Australian states [4,18].

In this study, we aimed to understand the role communication plays between key stakeholders (immunisation team members, school staff members who facilitate the immunisation clinics (hereafter ‘school coordinators’), parents, and adolescents) in contributing towards differences in vaccination coverage between schools in South Eastern Sydney. Communication is understood in this paper in a broad sense. It includes both written vaccine information and education, as well as information concerning the logistics of operating the school-based program, including stakeholder collaboration and engagement about the timing, consent, and running of the school clinic. Fieldwork for this study was conducted post-COVID-19 pandemic, during a period when the HPV dosage was reduced from two to one dose and during the transition to an online consent system in NSW schools.

## 2. Materials and Methods

### 2.1. Study Design

This study used a qualitative research design. We conducted semi-structured interviews with school coordinators, and parents of vaccine-eligible adolescents aged 12–13 years who were not fully vaccinated at school (had not received both dTpa and HPV vaccines). We also conducted a focus group with the immunisation staff.

### 2.2. Study Population and Sample

Fieldwork was conducted with three stakeholder groups. Senior immunisation staff working at SESLHD were purposively selected and five staff participated in an online focus group. Purposive sampling was used to recruit school coordinators from eleven schools with different vaccination coverages and a range of socioeconomic and demographic characteristics. The research team invited school coordinators to participate in this study via email, and interviews were scheduled at a mutually convenient time.

The participating school coordinators assisted in recruiting 11 parents for this study. Parents were included in this study if they had a child in Year 7/8 who had not received two vaccinations at school. School coordinators were given a list of students who were fully or partially vaccinated. Using this information, they determined which students were unvaccinated or undervaccinated and provided parents with an invitation to participate template. The invitation contained a link to an online research landing page hosted by REDCap (Vanderbilt, Nashville, TN, USA) and contained the Participant Information Statement and an electronic consent form. Individual school coordinators distributed the invitation via email and/or text message and were encouraged to follow up with a reminder message or phone call. Parents who consented to participate in this study completed the consent form and indicated their availability for an interview. The research team then followed up directly with these parents to schedule and conduct the interview.

### 2.3. Data Collection

Semi-structured interview guides were developed for each stakeholder group (immunisation staff, school coordinators, and parents) by the lead investigator (L.M.) and reviewed by all the authors. While the open-ended questions and prompts guided the interviews and focus group, the interviewers were flexible and iterative in their approach [19]. The topics for discussion with the school coordinators included the key factors that influenced the implementation of the school vaccination program and ongoing challenges. Both these issues were discussed with the immunisation staff, together with questions about the characteristics of schools with high and low vaccination rates and the perceived impact of sociodemographic factors on vaccination rates. The parent interviews explored views about vaccination in general; experiences with the school vaccination program; and barriers and facilitators of the program, including consideration of the communication strategies.

The interviews were undertaken between July 2022 and January 2024 and averaged 35 min in length (range 14–57 min). Interviews were conducted by L.M. or A.Y. Ten of the eleven parent interviews were conducted online or via phone and one written response was received. Eight interviews with school coordinators were conducted online and three face-to-face. The schools and parents received gift cards for their participation. The online focus group was conducted by C.D. and lasted 86 min. The focus group and interviews were recorded and transcribed verbatim, and the researchers wrote fieldnotes following the interviews and focus group. Data collection with immunisation staff and school coordinators stopped when data saturation was reached [20,21] and parent data collection ceased when recruitment yielded no further parent participants.

### 2.4. Data Analysis

The participant transcripts were coded in NVivo 12 (Lumivero, Denver, CA, USA). Deductive and inductive methods were used to code the data [22,23]. A.Y. and L.M. used a coding framework developed by C.D., which was reviewed in relation to our interview schedules and inductively adapted during the coding process [11]. A.Y. and L.M. coded the transcripts and compared several transcripts to ensure coding consistency across the data set. L.M. and A.Y. discussed and developed themes that arose from the data, which were further explored with C.V.-D. and C.D. to ensure all the themes were captured in the findings.

The socio-ecological model [24] guided the thematic analysis and facilitated the identification of communication barriers and facilitators for different stakeholders in the school vaccination program in South Eastern Sydney. This framework considers human behaviour in the context of interacting factors operating at different levels, including individual, interpersonal, organisational, and community and public policy factors impacting how vaccination and the school vaccination program are understood. The use of this model allowed for vaccination communication to be considered in terms of the stakeholders involved and the broader implications for the delivery of the school-based vaccination program [14,24]. The findings are reported using the Consolidated Criteria for Reporting Qualitative Research (COREQ) [20].

## 3. Results

### 3.1. Immunisation Staff and School Coordinator Demographics

Five key immunisation staff participated in the focus group and represented the following roles in the South Eastern Sydney public health unit: immunisation manager, administration officer, and three immunisation team leaders.

We undertook 11 interviews with school coordinators across school sectors. Twenty-eight schools were invited to participate, and seventeen schools declined or did not reply. Seven interviews were undertaken with coordinators in government schools, two in independent schools, and two in Catholic schools.

### 3.2. Parent Demographics

Eleven parents participated in this study. Ten completed interviews and one provided a written response. All parent participants were mothers, who had five female and six male adolescents between them. Based on the parental reporting of vaccination status, five children remained unvaccinated; three were partially vaccinated at school, specifically dTpa only and not HPV; and three students were fully vaccinated by their general practitioner (GP). Ten parents had completed high school, with eight holding university degrees and two with post-school qualifications. One parent had completed year 10 at high school.

### 3.3. Communication Barriers to Vaccination in the School Vaccination Program

We found a number of communication barriers and facilitators that impacted how the school vaccination program operated in South Eastern Sydney schools (Table 1). The findings are reported through the lens of the socio-ecological model [24] and are described according to how the individual, interpersonal, and organisational factors impacted the delivery of the school vaccination program.

#### 3.3.1. Low Parent Recall of Vaccination Information

The parents were asked to comment on the vaccination information provided by their school about the school vaccination program. With paper consent forms, schools provided parents with information kits sent home via students. Most parents reported receiving the school vaccination information via their child, but some did not receive it, as one parent noted:

*“So, I wouldn’t have gotten any note from her bag at any point in time. Let’s just say, notes in bags don’t work.” (Parent 3, adolescent fully vaccinated at GP)*

With the transition to online consent, the parents were emailed the vaccination information links. The following comments show that the recall of information delivered via hardcopy or online was low. When questioned further about the vaccination information, parents were unable to comment or recall anything about the content of the information:

*“Well so, yeah, I remember getting all that stuff. But, I just was like, yep. But I did ignore a lot of it.” (Parent 4, adolescent fully vaccinated at GP)*

*“There might have been a leaflet, but I don’t remember a kit, or anything else… I probably kept it for a while and then it just slipped my mind… No, I didn’t go into any of the links.” (Parent 6, adolescent unvaccinated)*

Some parents were unaware of which vaccinations their child was receiving, and while they may have received information or a link to vaccination information, the majority said they had not read the information, as noted by one school coordinator:

*“I think a lot of parents probably don’t even read it. Because often they ask, what is the vaccination? And like it’s on the information that goes home in the consent card, it’s in the email that I send them. I send them links to the New South Wales site and everything so they can get further information.” (School Coordinator 1)*

#### 3.3.2. School Staff Unaware of Consent Numbers

During fieldwork for this study, the vaccination consent process transitioned from paper to online. During the transition period, both paper and online consent were available for parents to complete. School coordinators reported that with the paper consent system, they had complete oversight over the number of students that returned parental consent for vaccination. This enabled a targeted approach to following up students prior to vaccination day and encouraging consent form return.

With the introduction of online consent, school coordinators reported they could no longer track which parents had consented to vaccination. Therefore, they were unable to directly target students who had not returned consent forms with communication reminders:

*“There was a lot of admin with the paper ones, because I would file them… I would tick them off, and I had to keep a file alphabetically so I could check who was missing, and we would chase people up… With the electronic one, I don’t know who has given consent until I get the list from Health.” (School Coordinator 4)*

#### 3.3.3. No Communication Channel Between Health Staff and Parents

At an interpersonal level, public health staff dealt directly with all schools in South Eastern Sydney to manage and administer the school vaccination program. This process was similar to how other health districts operate the school vaccination program in the Australian state of NSW. The immunisation staff interviewed for this study told us how they liaised directly with the immunisation coordinator in each school to arrange the logistics for clinic day. They reported that each school is then responsible for communication with students and parents and how consent for vaccination is communicated in the school community. We found no direct communication channel or relationship between the health staff that administered the vaccination program and the parents who consented to their adolescent’s vaccination. Without a direct connection to parents, the health staff reported they had minimal interaction with them and were unable to highlight the benefits of school vaccination for their adolescent. One school coordinator suggested that opening a communication channel between health staff and parents could help ensure that parents are well informed about the vaccination programs and encourage them to consent to vaccination for their adolescent:

*“I guess on parent information night, if you could have someone talk about it [vaccination] for a few minutes… a lot of the information is there, it is just getting them to read it, but if you’ve got the forum, especially with Year 7 for their information night, and you wanted to talk to them… because I think with a lot of them it is just understanding why and no, it is not COVID, and no, it is not an immunisation against boys.” (School Coordinator 8)*

#### 3.3.4. School Focus on Vaccination Logistics Rather than Education

The school coordinators overwhelmingly described a lack of dedicated time for immunisation coordination, forcing them to integrate vaccination tasks into their existing responsibilities. At an organisational level, we found that most school coordinators perceived their role as largely to facilitate the school vaccination program rather than to educate students and parents about vaccination. Following liaison with the health staff, the school coordinators communicated with parents about the practical details of school vaccination. They communicated, for example, the date of the vaccination clinic, passed on the vaccination information supplied by the South Eastern Sydney Health District, and gave details of how to consent for vaccination. Most school coordinators focused on sending consent reminders to parents to ensure their adolescent could be vaccinated in the school vaccination clinic, as highlighted by the following:

*“As I said, I am more of an administrative in this role; I see it more as an administrative role than as an educative role.” (School Coordinator 4)*

*“So in the end of last year we put one [newsletter] out saying from 2023 onwards it’s going digital, and there was an infographic about that… I don’t even actually think it says what the vaccine is and what it does or anything. I think it just says this is the name of the vaccine, Year 7 get it. This is the name of the Year 10 one, Year 10 get it. So that’s sort of the only thing we gave out and, no, I don’t think there’s any other like education as such as to what the vaccine is or what it prevents.” (School Coordinator 6)*

A few school coordinators reported outreach efforts to discuss the topic of vaccination with their students. These activities were undertaken by school coordinators, whose substantive roles were often as science or physical education teachers and who felt comfortable providing vaccination information and education. One school coordinator said they spoke in class about the importance of vaccination for health and wellbeing, emphasising the benefits of student involvement in the school vaccination program:

*“So, I just made really interesting PowerPoint slides that go on for 10 minutes, like what is meningococcal disease, why do you need to get vaccinated against it, what are the symptoms of the disease, and so on. And then I do the same with Year 7.” (School Coordinator 10)*

This was, however, rare, with school coordinators presenting the dominant view that the school provided the venue for vaccination and the coordinator’s role was to promote information about the vaccination clinic and secure consent for the vaccination to proceed, as highlighted by this coordinator:

*“I haven’t been informed that they’ve had an open discussion about vaccinations ‘cause most of the time I’ve noticed—with the Year 7s and the Year 10s—they ask a lot of questions on the day; “What’s this vaccination for? How many do we need to have?” and all that type of stuff. So I would say no, it’s not openly spoken about.” (School Coordinator 7)*

For the small group of coordinators who saw their role as incorporating vaccine information and education for students and parents, they were cautious not to be perceived as overstepping boundaries or pressuring parents. These coordinators were cautious to avoid conflict with parents with differing views on vaccination, as outlined by School Coordinator 6:

*“I think post COVID it’s a hard one because I think there is that sort of resistance and also that sort of people are tired of getting told they need to get vaccinated and that sort of thing.”*

#### 3.3.5. Limited Communication About Catch-Up Vaccinations

When students were absent from school on the vaccination clinic day, the health staff reported that they could “be caught up” or receive NIP-funded vaccinations at the next scheduled school clinic. While the health staff were clear about catch-up opportunities, at an organisational level, school coordinators, other school staff, and parents were less clear about this process.

Several scenarios highlighted the mixed messages around the catch-up process for missed vaccinations. Some school coordinators and reception staff told parents that they should organise a vaccination appointment with their general practitioner if they missed the school clinic:

*“…but admin will say, “I just told them to go to their GP.” And I’m like, “Okay, well that would be one option, but the second option is next time when they call say, look, we’re running another clinic on the day in this month, we’ll bring the card out and we can vaccinate them then.” (Focus Group)*

There was little understanding that students who had received parental consent for vaccination but missed the clinic were eligible to be vaccinated at the next school clinic. The parents also lacked awareness that catch-ups were an option. Several parents reported that because they had missed the school vaccination clinic, they believed they were responsible for organising vaccination:

*“Probably the GP. I’m not sure if the school offers it again. I don’t know if that’s another program that’s coming up in the future.” (Parent 8, adolescent partially vaccinated at school-dTpa only)*

Communication was also found to be absent regarding reminding parents who had not consented in the first instance, that another opportunity existed for parents to consent to vaccination. There was limited recognition that their child could be vaccinated at the next clinic, as highlighted by a parent and a school coordinator.

*“The school has a portal as well, and there might have been names of kids that had to go for the vaccine, but [my son’s] name wasn’t on there, so maybe that’s because I didn’t return the consent form, yeah, but I didn’t want to return the consent form, because I didn’t want him to have it, and I didn’t realise that I should’ve returned the consent form so he could have it later on.” (Parent 6, adolescent unvaccinated)*

*“Q: So, what’s the advice for those that miss out on vaccination day?*

*A: They can go to their local doctor and it’s free. So that’s the other thing. Sometimes they think they have to pay for it. So, I have to put in my things that I send out that it is a free vaccination provided by the Department.” (School Coordinator 9)*

Some school coordinators were also unsure about the catch-up process themselves: “But if we’re doing catch-ups, sometimes I don’t know who’s getting done” (School Coordinator 2), and another said: “I think just clear communication from New South Wales Health to us about the catch-ups that comes earlier the better. That’s really helpful because then I know how many kids are involved and I can get that information to parents sooner.” (School Coordinator 1).

Another parent commented on the school’s lack of warning about catch-up vaccinations. One mother reported her child’s experience of a catch-up vaccination. She said her child had received no warning about the vaccination, and it felt like it had been sprung on them. The end result was that the adolescent rejected the vaccination:

*“I totally forgot they were going to do the catch-up ones on that day, so I sort of just sent him and I didn’t even tell him about it. So, they called the Year 7 students up, that were in Year 7, to get them done and in Year 8, and I think he was sort of taken by surprise.” (Parent 7, adolescent unvaccinated)*

### 3.4. Facilitators for Vaccination in the School Vaccination Program

#### 3.4.1. Benefits of Using Established Parent and Student Relationships for Vaccine Communication

Several communication elements were reported as having facilitated involvement in the school vaccination by participants in this study. At an individual level, the health staff reported how school coordinators played a key role in how the immunisation program operated in each school. The attributes of the school coordinator, such as being well organised and motivated, were recognised as essential ingredients in how information about the vaccination program was communicated to the various stakeholders, including the school executive, other teachers and school staff, students, and parents. One immunisation team member reported the following:

*“…because the schools hopefully have a good, trusting relationship with parents—if the information is coming from them, I would hope it would be well received, and then the parents would be more likely to say, okay, this is something that will suit me, I can send this card to school, I can get my child vaccinated.” (Focus Group)*

Both immunisation staff and school coordinators recognised how important it was for school coordinators to use their established relationship with parents and students to communicate information about the upcoming school vaccination program. School coordinators noted that the established relationship with students meant they could target efforts to remind students about the vaccination clinic both before the day and on the day, as noted by this coordinator:

*“I think, yeah, making sure the kids know beforehand because it is needles, Year 7 kids especially, they are super anxious about it, there’s always one or two with tears, but just ensuring they have all the information that they need, and they know what’s happening on the day and how the day is going to run, so getting a timetable out to them so they know when in the day their vaccination is, because some are more nervous than others, so sometimes getting those kids through faster is better, yeah.” (School Coordinator 11)*

#### 3.4.2. Strong Communication and Relationships Between Health Staff and Schools

During the interviews, it was evident that a good rapport existed between the SESLHD staff and the coordinators at the individual schools who participated in this study. The school coordinators spoke highly of their positive interactions with the SESLHD immunisation coordinator and staff regarding setting up the clinic date and organising the practical aspects of delivering the school vaccination clinic. Interpersonal relationships were important, with the school coordinators reportedly being comfortable communicating with the health district staff and able to ask questions and clarify details about the clinic organisation:

*“We do work closely together, and my role requires me to have a close relationship with them in order to have a smooth clinic to be run, because the school knows the students more than us…so trying to tailor our service to each school.” (Focus Group)*

*“I think the way that they run that whole program is really, really solid… Things like using a calendar to put in your dates, doing the online learning. They did a few webinars about the new consent module. I’ve been so impressed with the level of communication and just how well things run, and how well organised they are. And that makes running the vaccinations a pleasure and really easy.” (School Coordinator 5)*

The health district staff stressed the importance of having engaged and well-supported school coordinators to the success of the school program. The health staff supported the school coordinators by providing pro forma emails to communicate with parents. The school coordinators commented on how useful these were in making communication with parents easier and more efficient:

*“So, I felt like everything that I needed was there. I didn’t really have to go back and forth and ask any follow-up questions or even parents didn’t even have to ask follow-up questions, which was great.” (School Coordinator 7)*

*“But I think this year the letters that were pre-written were a really good help. I was just pretty much like, even from the principal’s point of view, like he didn’t have to look at the wording for them and make sure it sounded good because he knew it was a department resource.“ (School Coordinator 6)*

The health staff were considered responsive by the school coordinators, as school coordinator 2 noted:

*“Look, I think they’re pretty good, they’re very quick to respond to questions. I’ve not ever had a problem. So, I think that’s doing a great job. So yeah, everything’s very clear and transparent.”*

In addition to the organisation of the program, the nurses who visited the school were also seen as important to the success of the school vaccination program and as having contributed to achieving high school vaccination rates, as noted by school coordinator 4:

*“Like the nurses are unreal, they’re so good with the kids, with the stress heads; they know how to keep them calm. Yep, I’ve always thought they’re fabulous, and I like working with the nurses we get out here, they are good.”*

At an organisational level, the health district and the education department were seen as working well together to roll out the vaccination program. While the school coordinators understood that the school vaccination program was the health department’s responsibility, they recognised that schools had a role to play in contributing to student health and wellbeing.

#### 3.4.3. Using Multiple Methods to Advertise Clinic and Consent Requirements

The school coordinators differed in their communication methods to promote the school vaccination date and the need for parents to consent to vaccination. Most used various written methods to communicate vaccination details with parents, including via emails, newsletters, school Facebook posts, and SMS messages:

*“I do the hard copy and the email, but I think the email gets more traction with families because it goes direct to the parent. The hard copy goes to the student and sometimes the student will lose it, not pass it on. It sits in their bag for a month that type of thing, whereas the email goes direct to parent. So yeah, you get more, I guess traction.” (School Coordinator 1)*

Some school coordinators used a mixture of written notes and digital communication via email, for example, one said the following:

*“We never send paper notes home ever anymore. It’s all email. So, something coming home on paper should be a good flag to them that there’s something important.” (School Coordinator 5)*

Some school coordinators targeted students with the information about the clinic date and the need for parent consent via written daily bulletins and verbal reminders in whole-school assemblies or communicated directly with the specific year group receiving vaccinations (Years 7 and 10):

*“Just constant reminders for our students, like our demographic needs that consistency. So, yeah, roll call every morning, in their year meetings every week, in assemblies every week. Just that consistency, that message, just being pumped through them during that period of time so that they are aware and they know that it’s coming… All the ones that I’ve said previously, I think all of them are needed to keep, like the consistent reminder. If the students are going home and telling their parents, then that’s what we need.” (School Coordinator 3)*

The school coordinators differed in when they started communicating with parents about the vaccination clinic and how often they sent reminders. Many started up to four weeks before the clinic date and sent reminders more frequently as the date drew nearer. School coordinators also said they tailored messages to make information more accessible to the diverse communities they served, including families from culturally and linguistically diverse, First Nations, and lower socio-economic backgrounds.

A few school coordinators mentioned that in addition to reminding students about the clinic date and obtaining parental consent, they undertook some student education about vaccination. These school coordinators saw the value of students understanding what vaccines were offered through the school-based program, what diseases they protected against, and why they should encourage their parents to consent to vaccination. This type of vaccination education was rare, and the school coordinators who did this were more likely to have substantive roles as science or personal development, health, and physical education (PDHPE) teachers and felt comfortable in their knowledge and ability to discuss vaccines and vaccination with their students:

*“She [Science Teacher] was great for giving out information, and there have been other schools too that have shown videos for both HPV and meningococcal ACWY.” (Focus Group)*

## 4. Discussion

We found several communication barriers that influenced the vaccination uptake in South Eastern Sydney schools. These included low recall by parents of vaccination information regardless of whether the information was delivered to parents in a hard copy or online. With the change from paper to online consent, school staff could not directly target communication with families who had not returned consent forms because they were unaware whether or not the online consent process had been completed. The lack of a direct communication channel was found to exist between health staff and parents, limiting opportunities for education and discussion with parents about vaccines. We also found there was limited communication within the school community about catch-up vaccinations for students who were absent/unable to be vaccinated on the clinic day. In terms of facilitators, the ability of schools to leverage already established relationships with parents to disseminate vaccination information and inform them about consent assisted in the vaccination process. Other facilitators included the strong rapport between health and school staff, where staff worked well together with good formal and informal communication channels, which resulted in students receiving vaccinations in a well-run clinic, and where schools used multiple methods for communication with the school community.

In the initial stages of this research, paper vaccination information was sent home via students, and following the onset of online consent, digital links to vaccination information were sent via email to parents. We found that parents either did not read or could not recall any specific information about the vaccine information delivered via either method. This finding is similar to another study [9], where the recall of HPV information provided to parents was low. Since information is key to parents giving informed consent for their adolescent’s vaccination, it may be worthwhile considering alternate communication strategies. Other methods outlined in the literature that could be considered by South Eastern Sydney Local Health District include using a social marketing campaign to engage and educate parents about the HPV vaccine [25] and enlisting parents in vaccination education programs [4]. Providing parents with accessible, culturally and linguistically diverse information is another consideration for South Eastern Sydney schools, which serve a culturally diverse population [26,27].

Participants reported in this study that schools in South Eastern Sydney focused on the logistics and delivery of the school vaccination program rather than emphasising the role of education and information in the rollout of the school program. While school coordinators explained how students relayed information to their parents about the date of the vaccination clinic and were often given multiple reminders to encourage parental consent, we found it rare for students to be actively involved in conversations at school about vaccination. In contrast to these findings, other studies [9,11] described the benefits of in-school vaccine education, which resulted in increased vaccine literacy, reduced adolescent vaccination-related anxiety, and a more positive adolescent vaccination experience. By making adolescents central to receiving quality information and education about vaccines and vaccination at school, there may be potential for them to develop vaccination literacy [12,13,28,29]. Improved adolescent vaccination literacy and effective communication between adolescents and their parents promotes shared vaccination decision making and may lead to a better vaccination experience for adolescents [11]. Furthermore, effective communication between adolescents and their parents about vaccination, combined with optimised program organisational strategies, may positively impact parental consent to vaccination [4,9].

The study participants recognised the key role played by school coordinators in the operation of the school program. Student education about vaccines was reported by school coordinators to often be informal in response to student questions and concerns. With our finding that the schools focused more on logistical arrangements about vaccine clinics rather than education, there may be scope to ensure that school coordinators and other staff members, including school administration staff, are adequately informed and confident in their responses. This may require additional training or resources to enhance their knowledge about vaccines and vaccination programs.

While the health staff in this study spoke about the availability of catch-up vaccines at school, the parent and school staff participants were uncertain about the vaccine catch-up process. The school participants and parents in this study reported limited awareness that the option existed for students to be vaccinated at subsequent school clinics. Communication within the school program could be strengthened to ensure school staff have this knowledge to communicate to students and parents, which could result in greater vaccine coverage. This is in contrast to assuming that students absent on vaccination day are required to make their own arrangements for vaccination outside of school by, for example, arranging a visit to a doctor or pharmacy. School staff also need to ensure that students without parental consent for vaccination are included in communication about upcoming vaccination opportunities.

To increase vaccination coverage in South Eastern Sydney, it would seem pragmatic to focus on addressing the barriers and strengthening the facilitators in new and different ways. Like in other studies, our findings highlight the importance of strong communication and relationships between health and school staff. Cuccaro et al. [25] highlighted the strong partnerships between health districts and participating schools as crucial for strong program implementation in schools. Dube et al. [2] also found that collaboration with school staff was essential to ensuring the school program was delivered in an efficient and effective way and noted the important role of school staff in ensuring parents are reminded about the vaccination clinic and encouraging the return of consent.

By using multiple methods of communication, teachers in this study sought to remind students and parents about the vaccination clinic. While participants mentioned mainly verbal or written reminders in this study, other communication methods used in other jurisdictions include using a printed banner placed in the school drop off area, reminding parents of the vaccination date and providing contact details for the nursing team [25], or the use of social marketing to foster vaccine acceptance among parents [30].

### Strengths and Limitations

The strength of the research design was that the use of interviews allowed for a greater depth of information to be captured, and hearing the views and opinions of parents with partially vaccinated students, which is not a group often included in research. There were some limitations in this study. Electronic consent was introduced during fieldwork and the HPV schedule changed from two doses to one dose, which impacted this study’s continuity. The COVID-19 pandemic also caused delays in commencing this study and impacted the recruitment, particularly of teachers and parents. Following the COVID-19 pandemic, schools experienced curriculum disruptions and high staff absences, which resulted in challenges to the recruitment of school staff. Furthermore, the research team relied on school coordinators to identify students who were not fully vaccinated and invite their parents to participate in this study, which was impacted by the challenges with recruitment of school staff. Lastly, this study was undertaken in a relatively small geographic location, so the findings may not be generalisable to other areas or jurisdictions.

## 5. Conclusions

This study investigated the communication barriers and facilitators in the operation of the school vaccination program in South Eastern Sydney. We found that communication barriers existed at the individual, interpersonal, and organisational levels. These barriers included the low recall of vaccine information, the absence of a communication channel between the health staff and parents, and a school focus on the vaccination facilitation and logistics over the vaccine education of the students and parents. The communication facilitators included the effective relationships between health and school staff and the use of multiple methods to inform parents about upcoming vaccination clinics. These findings suggest that targeted vaccine information and education strategies for students and parents could strengthen vaccination coverage in the district, as could developing a communication channel between parents and health staff. The well-established relationship between the health district and local schools could be leveraged to develop these initiatives. Future research is recommended to evaluate the efficacy of these interventions and to understand the impact of these communication strategies on the vaccine coverage in this location.

## Figures and Tables

**Table 1:** Summary of themes and sub-themes.

Theme	Sub-Themes
(1) Communication barriers to vaccination in the school vaccination program	Low parent recall of vaccination information Challenges in consent tracking No communication channel between health staff and parents Greater school focus on vaccination facilitation than student education Limited communication between stakeholders about catch-up vaccinations
(2) Communication facilitators to vaccination in the school vaccination program	Established school/parent relationships for vaccine communication Effective communication between health and school staff Using multiple methods to promote clinic and consent requirements

## Data Availability

The data presented in this study are available upon request from the corresponding author. Due to ethical restrictions, they are not publicly available.
